# Keeping up with the guidelines: design changes to the STREAM stage 2 randomised controlled non-inferiority trial for rifampicin-resistant tuberculosis

**DOI:** 10.1186/s13063-022-06397-4

**Published:** 2022-06-07

**Authors:** Ruth L. Goodall, Karen Sanders, Gay Bronson, Meera Gurumurthy, Gabriela Torrea, Sarah Meredith, Andrew Nunn, I. D. Rusen, on behalf of the STREAM Trial Team, Gay Bronson, Meera Gurumurthy, Jan Komrska, Leena Patel, Ishmael Qawiy, I. D. Rusen, Sonia Ali, Katharine Bellenger, Deborah Bennet, Rachel Bennet, Wendy Dodds, Ruth Goodall, Sarah Meredith, Brendan Murphy, Andrew Nunn, Carol Roach, Karen Sanders, Johanna Whitney, Armand Van Deun, Gabriela Torrea, Chen-Yuan Chiang, Laura Rosu, Bertie Squire, Jason Madan

**Affiliations:** 1 MRC Clinical Trials Unit at UCL, Institute of Clinical Trials & Methodology, University College London, 90 High Holborn 2nd Floor, London, WC1V 6LJ UK; 2 Research Division, Vital Strategies, New York, USA; 3 Vital Strategies, Singapore, Singapore; 4 Institute of Tropical Medicine, Antwerp, Belgium

**Keywords:** Trial, Design, Rifampicin resistance tuberculosis

## Abstract

Results from the STREAM stage 1 trial showed that a 9-month regimen for patients with rifampicin-resistant tuberculosis was non-inferior to the 20-month regimen recommended by the 2011 WHO treatment guidelines. Similar levels of severe adverse events were reported on both regimens suggesting the need for further research to optimise treatment. Stage 2 of STREAM evaluates two additional short-course regimens, both of which include bedaquiline. Throughout stage 2 of STREAM, new drug choices and a rapidly changing treatment landscape have necessitated changes to the trial’s design to ensure it remains ethical and relevant. This paper describes changes to the trial design to ensure that stage 2 continues to answer important questions. These changes include the early closure to recruitment of two trial arms and an adjustment to the definition of the primary endpoint. If the STREAM experimental regimens are shown to be non-inferior or superior to the stage 1 study regimen, this would represent an important contribution to evidence about potentially more tolerable and more efficacious MDR-TB regimens, and a welcome advance for patients with rifampicin-resistant tuberculosis and tuberculosis control programmes globally.

**Trial registration:** ISRCTN ISRCTN18148631. Registered 10 February 2016

## Introduction

Results from the STREAM stage 1 trial showed that a 9-month regimen for patients with rifampicin-resistant tuberculosis (RR-TB), based on the regimen described by Van Deun [1, 2], was only 1% less effective than the 20-month regimen recommended by the 2011 WHO treatment guidelines [3], a difference that satisfied the predetermined criteria of non-inferiority [4, 5]. The 9-month regimen was comprised of moxifloxacin (at higher than standard dose), clofazimine, ethambutol and pyrazinamide given for 40 weeks with kanamycin, isoniazid and prothionamide given during the 16-week intensive phase. Similar levels of severe adverse events were reported on both regimens. Whilst the non-inferiority of a shortened regimen was encouraging, the lack of benefit in either efficacy or safety suggested the need for further research to optimise treatment for RR-TB [5].

Before the results of stage 1 of STREAM were known, the trial sponsor and investigators were invited by the funder to consider the evaluation of regimens using new drugs. Stage 2 of the trial was subsequently launched to evaluate two additional short-course regimens, both of which included bedaquiline, a new drug granted a provisional license from the US Food and Drug Administration (FDA) in December 2012. The original design of stage 2 of STREAM has been outlined previously [6]. The 20-month regimen (denoted regimen A) was included in stage 2 since the non-inferiority of the 9-month regimen (regimen B) had not yet been established [6], thus avoiding the possibility of ‘bio-creep’, a phenomenon whereby a slightly inferior treatment becomes the active control for the next generation of non-inferiority trials leading to a decline in efficacy of treatments over time [7].

**Fig. 1:**
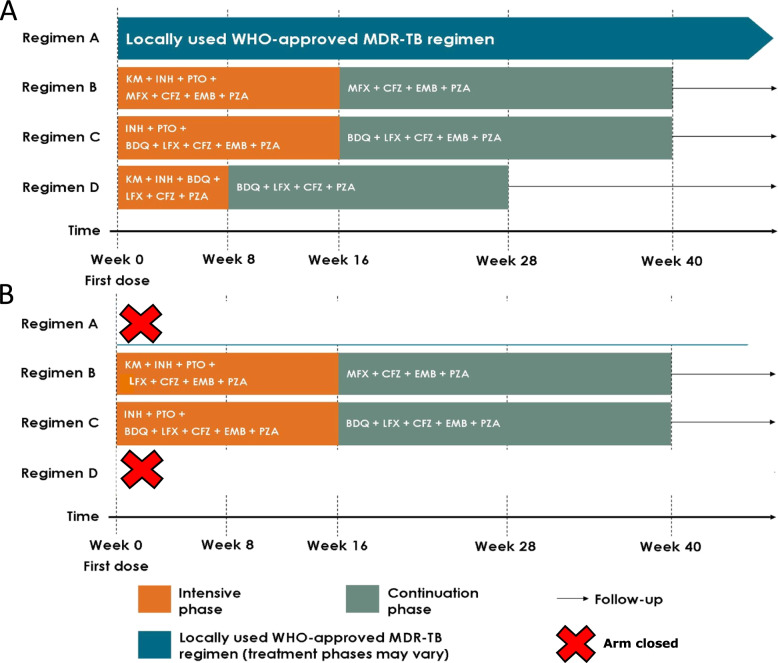
Randomised treatment regimens

STREAM regimen C is a 9-month oral regimen that is the same as regimen B, except that bedaquiline replaces kanamycin and is prescribed throughout the 9 months, and levofloxacin replaces moxifloxacin, as the combination of bedaquiline and moxifloxacin was contraindicated by the FDA because of the risk of QT prolongation. A comparison of regimens C and B therefore evaluates whether the challenges of administration and toxicity of the injectable drug in Regimen B can be removed whilst maintaining efficacy. STREAM regimen D was designed to explore whether treatments could be further simplified and shortened to 6 months; it consists of bedaquiline, clofazimine, pyrazinamide and levofloxacin prescribed for 28 weeks, supplemented by high-dose isoniazid with kanamycin for a short intensive phase of 8 weeks (Fig. 1A). All regimens include the option to extend the intensive phase by 4 or 8 weeks in the event of delayed sputum smear conversion. STREAM stage 2 is the registrational trial for bedaquiline and regulated by the FDA and the European Medicines Agency (EMA).

**Fig. 2:**
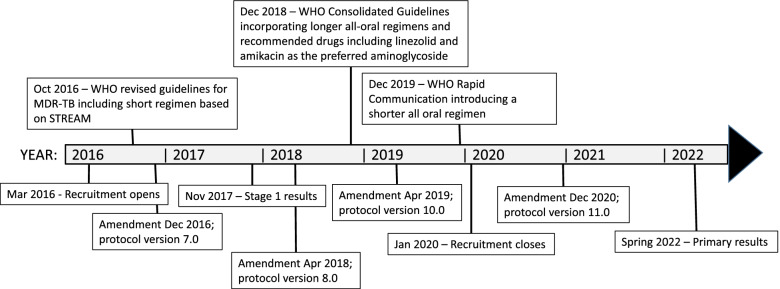
Timeline of changes to trial design and release of WHO treatment guidelines

Since STREAM stage 2 opened to recruitment in March 2016, the treatment landscape for RR-TB has been rapidly evolving. This paper describes changes to the trial design in response to new treatment guidelines and the evolving priorities of clinicians and patients; it outlines the four major protocol amendments between the initiation of stage 2 and the present (Fig. 2). The amendment dates reported correspond to the time of centralised approval; implementation at site level occurred at varying times, typically within the subsequent 12-month period. Resulting changes to the Statistical Analysis Plan for stage 2 are also discussed. Analyses of the primary endpoint for STREAM stage 2 are planned for 2022. Long-term efficacy and safety outcomes will be reported after 132 weeks of follow-up in 2023.

## Modification of trial regimens

After the release of the 2016 WHO treatment guidelines [8], the majority of countries began to adopt a regimen similar to STREAM regimen B as the standard treatment in their national tuberculosis programmes (NTP). In response, the protocol was amended (Dec. 2016, version 7.0) to permit sites that had implemented the WHO 2016 recommendations to cease randomisations to regimen A, as starting a 20-month regimen would have been inappropriate and unacceptable given the change to the standard of care. By 2018, in version 8.0 of the protocol, randomisation to Regimen A stopped altogether as all participating countries had moved (or had plans to move) to a shorter regimen by that time.

Randomisations to regimen D also ceased in protocol version 8.0. Recruitment to the trial had been slower than expected. In addition, interest in shortened regimens containing no injectable agents was increasing, with fully oral 6-month regimens under evaluation in a number of phase 3 trials [9, 10]. Regimen D had therefore become a less attractive treatment option than at the start of the trial, with a lower chance of use in NTP even if effective [11], and ceasing randomisation to regimen D would ensure the trial reached the sample size requirements for the comparison of regimens B and C, that being of primary importance.

Regimen B has also been modified since enrolment to stage 2 began. Results from STREAM stage 1 showed evidence of increased risk of QT prolongation on regimen B compared with regimen A [5]. Ten percent of participants experienced QT or QTcF prolongation over 500 ms, with no pattern to when an individual’s first severe QT prolongation might occur during the 9 months of treatment. Therefore, implementation of regimen B with clofazimine and high-dose moxifloxacin under programme conditions would require regular ECG monitoring throughout treatment. In light of this, moxifloxacin was replaced by levofloxacin in regimen B in April 2018 (protocol version 8.0) as levofloxacin was expected to have less effect on the QT interval. We will be able to assess whether this reduces the safety monitoring requirements of regimen B.

The cessation of randomisations to regimen D and change to the fluoroquinolone in regimen B were implemented at sites after local regulatory and ethics approval in all countries except India. The Central Drugs Standard Control Organization (CDSCO) headed by the Drugs Controller General of India (DCGI) did not approve these changes in protocol version 8.0, which were never implemented in India. Moxifloxacin had been approved as the fluoroquinolone to be used in the NTP for India not long before, and there was reluctance to move away from its use. In addition, there was still strong interest in regimen D. All other changes made in protocol version 8 were approved in India, as well as subsequent protocol amendments.

In April 2019 (protocol version 10.0), regimen B was further modified by the replacement of kanamycin with amikacin in sites where the NTP was using amikacin as the preferred injectable in line with revised WHO guidance [12], and amikacin was available for trial participants. In the event, it was not necessary to make this change in any of the trial sites. The regimens under randomisation from protocol v8.0 onwards are summarised in Fig. 1B.

As a consequence of these changes to the protocol, all analyses will be stratified by the protocol version in use at the time of randomisation (on an individual basis) to ensure all treatment comparisons are made to concurrent controls.

## Sample size

The initial sample size for stage 2 of 330 participants each on regimens B, C and D assumed a favourable efficacy outcome of 75% at week 76 in all three regimens, a 10% non-inferiority margin, that 10% of participants would not be assessable in the primary analysis, 80% power and 2.5% significance (one-sided).

After randomisations to regimen D ceased with protocol version 8.0, and the results of stage 1 became available, the sample size assumptions were revised. The assumed proportion of participants with a favourable efficacy outcome at week 76 on regimen B was estimated to be 80%, based on the stage 1 results. In addition, the assumption that there would be no difference in the point estimate for outcome in regimens B and C was revisited. This was considered unduly conservative and the assumptions were revised to an expected benefit in efficacy of 2% using 40 weeks of treatment with bedaquiline compared to 16 weeks of treatment with kanamycin (the only difference between regimens B and C after the replacement of moxifloxacin with levofloxacin in protocol version 8.0), that is to say an assumed favourable efficacy outcome at week 76 of 82% for regimen C. Based on these changes, with all other assumptions remaining the same, 172 evaluable participants would be required in each of regimens B and C. The sample size was therefore reduced, and the trial aimed to enrol 200 participants each to regimens B and C, which allows for up to 14% of participants excluded from the efficacy analysis in the per-protocol population.

A sample size re-estimation (SSR) procedure was included in the protocol at the time these adjustments to the sample size were made to check that the sample was sufficient. The SSR was to be performed when at least 40% of the 400 participants on regimens B and C had completed 24 weeks of treatment and at least 10% of the 400 participants had reached week 76. A pooled estimate of the week 76 favourable efficacy outcome proportion over the two treatment arms is obtained from a Kaplan-Meier analysis of the time-to-unfavourable event data of all subjects included in the interim analysis. A new sample size is calculated using this estimate and the protocol assumption of a 2% treatment difference in favour of regimen C. The sample size is increased to the new value (with an upper cap) if the estimate of the pooled favourable efficacy outcome proportion is below a pre-defined cut-off which was chosen to be 0.75 (see Appendix).

In March 2019, the SSR procedure was followed with the cumulative probability of favourable outcome in regimens B and C combined by 76 weeks estimated as ≥ 75%. Per SSR decision rules, the TSC decided that enrolment should stop when at least 200 participants had been randomised to each of regimens B and C. Randomisation ended in January 2020, when 202 and 211 participants had been randomised to regimens B and C respectively.

## Objectives/outcomes

The trial currently has one primary objective, to assess whether the proportion of participants with a favourable efficacy outcome on regimen C is non-inferior to that on regimen B at 76 weeks. The co-primary endpoint assessing the non-inferiority of regimen D compared to regimen B was made a secondary objective in protocol v8.0 after the decision was made to halt randomisations to regimen D. Implementation of protocol v8.0 was not immediate across sites however, and, combined with the continued randomisations to regimen D in India, a total of 143 participants were randomised to regimen D, making informative comparisons between regimens B and D possible. Another original co-primary objective, assessing the superiority of regimen C compared to regimen B, also became a secondary objective in protocol version 8.0 due to reduced power. In light of the change of fluoroquinolone in regimen B implemented in protocol version 8.0, separate secondary efficacy objectives were added to compare regimen C to regimen B containing moxifloxacin and to regimen B containing levofloxacin.

The primary outcome is a composite endpoint combining unfavourable outcomes related to bacteriological and clinical events, including treatment changes due to failure or toxicity. Treatment changes that constitute an unfavourable efficacy outcome were also modified as the trial progressed. Since the start of stage 2, initiating bedaquiline when the originally allocated regimen did not include it has been considered an unfavourable event. In version 7.0 of the protocol (December 2016), the definition of unfavourable outcome was updated to clarify that if kanamycin (or another injectable agent) was added to the regimen of a participant on the fully-oral regimen C, this would also be considered unfavourable. This change was made to balance the most likely treatment changes for Regimens B and C. After publication of the revised WHO guidelines in 2018 [13], in which it was recommended that bedaquiline, linezolid, and either moxifloxacin or levofloxacin should be used in all MDR treatment regimens and kanamycin should be avoided, it became more likely that a treatment change for those initially allocated to regimen C would be to add linezolid, rather than kanamycin or another injectable agent should bedaquiline have to be stopped. Therefore, initiating linezolid was added as an unfavourable outcome in version 11.0 of the protocol, which was approved in December 2020.

There will be two sensitivity analyses of the primary outcome in light of the changes to regimen B (as outlined above) and the addition of linezolid to the single drug changes that constituted an unfavourable event. These will ignore (i) substitutions of levofloxacin for moxifloxacin and vice versa if the fluoroquinolone is changed and (ii) starting linezolid alone, within the definition of an unfavourable event, respectively.

Following additional exploratory analyses undertaken in stage 1, a new secondary outcome assessing time to failure or recurrence has also been added, the aim of which is to evaluate the comparative merits of each regimen focusing only on TB-disease outcomes (ignoring treatment changes) [14]. For this outcome, a prospective assessment of the probability that a participant was failing to be cured or was experiencing TB recurrence at the time of their primary endpoint is made by an independent clinician masked to treatment allocation. Participants are assigned to one of 5 categories depending upon their likelihood of being a failure or recurrence: definite, probably, possibly, likely or highly unlikely. For analysis, participants in the definite and probably categories are considered to have evidence of failure or recurrence at the time they are classified as unfavourable according to the primary outcome, with all other participants censored at the time of their primary endpoint. Standard time to event analyses are then carried out.

## Duration of follow-up

The primary outcome for stage 2 is assessed at 76 weeks from randomisation (36 weeks after completion of treatment for regimens B and C, and 48 weeks for regimen D). This is expected to capture most relapses that occur post-treatment, based on evidence from stage 1 of STREAM and trials in drug-sensitive disease [1, 15].

The initial time-point for assessment of long-term efficacy and safety outcomes was 132 weeks from randomisation. In the current protocol (version 11.0), the timing of assessment of long-term safety was unchanged, but assessment of efficacy was brought forward to the date on which the last participant recruited reaches 96 weeks of follow-up (30 November 2021). Prior to implementing this change an assessment of the impact was made. The trial team concluded that the impact of shortening the follow-up period for efficacy would be minimal (as measured by loss of person-years of follow-up) and outweighed by the benefits of earlier availability of long-term trial results. In most participants (approximately 75% on regimens B and C), long-term efficacy will be assessed at week 132; the remainder (those randomised towards the end of the recruitment phase) will have a reduced follow-up for efficacy to between 96 and 132 weeks. The reduction in the total person-years of follow-up for the efficacy analysis is small, only 3% in regimens B and C.

Given the shorter efficacy follow-up in one quarter of participants, the proportion of participants with a favourable efficacy outcome at week 132 will now be estimated using time to unfavourable outcome and the Kaplan-Meier product-limit estimator, thereby using information on all eligible participants. Data from participants whose scheduled last efficacy visit is before week 132 will be censored at the time of their last visit, unless they have already become unfavourable. The secondary objective assessing the non-inferiority of regimen C to regimen B will be analysed using a stratified risk difference, calculated with confidence intervals based on bootstrap standard errors. A sensitivity analysis will estimate the proportion of participants with an unfavourable outcome at Week 132 on the subset of participants randomised at least 132 weeks on or before the date of the last efficacy visit (30 November 2021).

The follow-up for long-term safety remains unchanged at 132 weeks because of the importance of obtaining maximum information on mortality, a key safety outcome. In the C208 phase 2b trial of bedaquiline significantly more deaths were observed on the bedaquiline containing arm than in the control arm regimen; many of these which were long after the end of treatment, despite better microbiological outcomes in the former [16]. The reasons for the observed increase in overall mortality is as yet unclear and may well have been due to chance; the results from STREAM will provide more information on this important question.

## Changes due to the COVID 19 pandemic

The first wave of the COVID-19 pandemic occurred just as recruitment to stage 2 had been completed. In March 2020, over 80% of participants had finished their trial treatment, but 75% of participants were still in follow-up. Although sites experienced lockdowns and other restrictions on movement to address the pandemic, site staff were able to ensure participants received all prescribed treatment and adequate safety monitoring through a combination of remote, at home and in-clinic follow-up visits. The impact on completion of treatment, follow-up data collection and availability of microbiology results at weeks 76 and 132 has been minimal.

Protocol changes were made in December 2020 (version 11.0) to extend the boundaries of the week 76 and week 132/last efficacy visit windows (previously ± 6 weeks) for any stage 2 participants whose appointment was scheduled to occur during the COVID-19 pandemic and did not occur due to restrictions on movement, unacceptable risk of exposure to COVID-19 in connection with the scheduled visit or any other reason related to the pandemic. For these participants, the week 76 sputum samples must now be taken in a window beginning six weeks prior to the scheduled visit date and ending within the week 84 visit window, i.e. within 14 weeks of the scheduled week 76 visit date. Similarly, sputum samples for the last efficacy visit must be taken in a window beginning 6 weeks prior to and up to 12 weeks after the scheduled visit date (an extension of 6 weeks).

Participants with no sputum sample available within the revised week 76 window due to COVID-19 restrictions will be considered unfavourable. Sensitivity analyses will reclassify these participants as (i) non-assessable and excluded from the primary efficacy analysis and (ii) favourable if they meet the definition of favourable with the latest of the 2 negative culture results being within the week 68 window and unfavourable otherwise.

## Other changes

The eligibility criteria for stage 2 have been amended slightly since the start of the trial. In December 2016 (version 7.0), the minimum age of participants was lowered from 18 to 15 years old, with the aim of increasing the generalisability of the trial results. From April 2018 (version 8.0), anyone GeneXpert positive with a cycle threshold below 25 was eligible for recruitment regardless of their HIV or smear status. In April 2019 (version 10.0), an additional exclusion criterion was added following a safety update by the EMA, making anyone who had previously experienced a serious adverse reaction when taking a quinolone ineligible for entry to the trial. As version 10.0 was not implemented in India, sites were provided with the safety letter from the EMA as an update and for notification of their Ethics Committees.

Safety monitoring was simplified in protocol version 8.0 (April 2018), with a move from triplicate to single ECGs, as experience up to this point indicated a single ECG would be sufficient. If QTcF prolongation of 500 ms or more was detected, then two further ECGs were collected. The requirement for routine laboratory safety tests after week 76 was ended and laboratory safety tests required only if clinically indicated.

## Conclusion

STREAM stage 1 demonstrated the efficacy of a shortened, 9-month regimen for RR-TB, which the WHO recommended as an option for the standard care of MDR-TB in December 2018. Nevertheless, similar levels of severe adverse events were reported in both regimens during stage 1, suggesting the need for further research to optimise RR-TB treatment. Stage 2 builds on the experience from stage 1 to evaluate two new bedaquiline-containing short-course regimens—an all-oral 9-month regimen and an injectable-containing 6-month regimen.

Throughout stage 2 of STREAM, new drug choices, evolving global treatment guidelines, and a rapidly changing treatment landscape have necessitated changes to the trial’s design to ensure it remains ethical and relevant. The design changes discussed above, with the corresponding changes to the Statistical Analysis Plan, help ensure that stage 2 continues to answer important questions. It will contribute to the growing body of evidence regarding the safety of bedaquiline and provide data on the efficacy and safety of a 6-month regimen for RR-TB. If the STREAM experimental regimens are shown to be non-inferior or superior to the stage 1 study regimen, this would represent a welcome advance for patients with RR-TB and TB control programmes globally.

## Data Availability

Not applicable.

## Appendix

### Sample size re-estimation: choice of cut-off

A simulation-based evaluation (100,000 replicates per scenario) of the performance of the SSR procedure was performed. The cut-off for the estimate of the pooled favourable efficacy outcome proportion below which a sample size increase would be implemented was chosen to be 0.75. The choice of this cut-off was based on the criteria that when the protocol assumptions hold true, the probability of deciding to increase the sample size is low (~ 5%). On the other hand, when the favourable efficacy outcome proportion in the control arm is low, and a sample size increase would be warranted to preserve the power of the study, then the probability to increase the sample size is high (> 80%). Applying this decision rule in the simulation study, assuming a difference between regimens of 2% in favour of regimen C the power was estimated to be > 80% for all pooled favourable efficacy outcome proportions > 60%. Power was reduced if the assumed difference between regimens was 1% or 0% (to > 70% and > 65% respectively). The type I error rate of the SSR design was well controlled; simulation results showed a Type I error inflation of at maximum 0.06%.

## Abbreviations

EMA: European Medicines Agency
FDA: US Food and Drug Administration
HIV: Human immunodeficiency virus
MDR-TB: Multidrug-resistant tuberculosis
NTP: National Tuberculosis Programmes
QTcF: QT interval corrected for heart rate by Fridericia’s formula.
RR-TB: Rifampicin-resistant tuberculosis
SSR: Sample size re-estimation
WHO: World Health Organisation
